# Osseous Bridges of the Sphenoid Bone: Frequency, Bilateral and Sex Distribution

**DOI:** 10.3390/biology12040492

**Published:** 2023-03-24

**Authors:** Silviya Nikolova, Diana Toneva, Dora Zlatareva, Nevena Fileva

**Affiliations:** 1 Department of Anthropology and Anatomy, Institute of Experimental Morphology, Pathology and Anthropology with Museum, Bulgarian Academy of Sciences, 1113 Sofia, Bulgaria; 2 Department of Diagnostic Imaging, Faculty of Medicine, Medical University of Sofia, 1431 Sofia, Bulgaria

**Keywords:** caroticoclinoid foramen, interclinoid bridge, pterygospinous bridge, pterygospinous (Civinini’s) foramen, pterygoalar bridge, pterygoalar (Hyrtl’s) foramen

## Abstract

**Simple Summary:**

The sphenoid bone occupies the central part on the skull base and forms the floor of the middle cranial fossa where some important foramina-transmitting neurovascular structures are placed. Usually, there are few ligamentous connections between definite parts of the sphenoid bone, which occasionally may be replaced by bone formations. These bone bridges form additional foramina, which could compress the passing neurovascular structures. Sphenoid bridges also have a significant impact on the regional neurosurgery since they obstruct the surgical corridors to some regions of the skull base. In this study we investigate the frequency, bilateral and sex distribution of the different types of sphenoid bridges in Bulgarians. The obtained results show that the osseous bridges of the sphenoid bone are relatively common findings in the investigated series. This should be kept in mind during diagnostic and subsequent treatment of some conditions, which could be induced or related to the replacement of usual ligaments of the sphenoid bone with osseous bridges.

**Abstract:**

Sellar (caroticoclinoid and interclinoid), pterygospinous and pterygoalar bridges are osseous bars of the sphenoid bone, which enclose additional foramina in the skull base and could cause entrapment of nerves, occlusion of vessels and obstruction of surgical corridors. This study aimed to investigate the frequency of sphenoid bone bridges in Bulgarians and to assess the bilateral and sex differences in their distribution. This study was performed on head CT scans of 315 Bulgarians, 148 males and 167 females. The sellar bridges were the most common type of sphenoid bridging; particularly the caroticoclinoid bridge. The pterygospinous bridge was a relatively common finding and the pterygoalar bridge was the most infrequent type of bridging. The total frequency of sellar bridges did not differ significantly between both sides and sexes. The pterygospinous bridge did not indicate significant bilateral differences but showed considerable sex differences concerning the left-side occurrence, which was significantly higher in the male series. There were no considerable bilateral and sex differences in the distribution of the pterygoalar bridging. There were no significant correlations between the different types of sphenoid bone bridges, but each type of bridging showed significant positive correlations between the right and left side co-occurrence in males and females.

## 1. Introduction

Bridges of the sphenoid bone are osseous bars, which enclose additional foramina in the cranial base. When presented, these bone bridges interact with the surrounding neurovascular structures, which makes them of clinical importance. Moreover, the sphenoid osseous bridges and the resultant additional foramina exert a serious impact on the neurosurgery in the parasellar area [1,2] and the region of the infratemporal fossa [3,4] since they alter important landmarks and increase the risk of intraoperative injuries [1,4].

The sellar bridges (SB) interconnect the clinoid processes (anterior, middle and/or posterior) of the sphenoid bone and form additional foramina. A bone connection between the anterior and middle clinoid processes forms a cariticoclinoid bridge (CcB). The CcB encloses the so-called caroticoclinoid foramen (CcF), clinocarotid canal or anterior interclinoid (arterial) foramen, which makes a passage for the internal carotid artery (ICA). A common osseous bridge connecting the anterior, middle and posterior clinoid processes forms a CcF anteriorly and a posterior interclinoid (venous) foramen posteriorly. The posterior interclinoid foramen gives way to the lateral part of the circular sinus. An osseous bridge between the anterior and posterior clinoid processes, the so-called interclinoid bridge (IcB), in the absence of a CcF, encircles a common interclinoid foramen (canal of Gruber); it transmits the ICA anteriorly and the lateral part of the circular sinus posteriorly [5]. 

The pterygospinous bridge (PsB) is located between the angular spine of the sphenoid bone and the spine of Civinini, situated at about the middle of the posterior border of the lateral pterygoid plate. The PsB encloses the Ps (Civinini’s) foramen (PsF), which varies in size not only in different skulls but on the two sides of the same skull. The axis of the Ps foramen is vertical, and it lies either below or on the medial side of the foramen ovale [3]. Branches of the mandibular nerve and vessels may pass through the PsF to the medial pterygoid muscle and to the tensors tympani and veli palatini [3,6]. In general, the PsB affects the distribution pattern of the mandibular nerve as it passes through the foramen ovale and causes it to begin division into its main branches to the temporalis, masseter and pterygoid muscles. Their course could be either lateral or medial towards the PsB. The lateral course of the branches except for the chorda tympani is the most common pattern, whereas the medial course of all mandibular nerve branches is the least frequent pattern [4]. 

The pterygoalar bridge (PaB) connects the inferior surface of the sphenoid greater wing, near the anterolateral edge of the foramen spinosum, and the root of the lateral pterygoid process. The PaB forms the pterygoalar foramen (PaF) also known as porus crotaphitico-buccinatorius and Hyrtl’s foramen. The axis of the PaF is horizontal and lies on the lateral side of the foramen ovale. However, the PaB may also run beneath the foramen ovale, dividing it into two parts [3]. A number of branches of the mandibular nerve could pass through the PaF. Among them are the nerve to the buccinator muscle, the nerve to the lateral pterygoid muscle and a nerve or nerves to the temporal muscle. Sometimes the nerve to the masseter muscle may also pass through this foramen or canal [3,6]. Some veins of the pterygoid plexus and a few small arteries may also pass through it [3]. 

The osseous bridges of the sphenoid bone interact with the nearby neurovascular structures and may potentially cause complications of heterogeneous origin, which makes them of significant importance in clinical practice. The aim of this study is to investigate the frequency of the different types of sphenoid bridges in contemporary Bulgarians, and to assess the bilateral and sex differences in their distribution. 

## 2. Material and Methods

The sample included 315 Bulgarians (148 males and 167 females) aged between 13 and 94 years with an average age of 53 years (males 16–88, average 51 years; females 13–94, average 54 years). This study was performed on head CT scans generated using a CT system Toshiba Aquilion 64. The scanning parameters were tube voltage of 120 kV, tube current ranging from 165 to 500 mA, exposure time of 0.5 s, and detector configuration of 32 × 0.5 mm. The protocol of image reconstruction incorporated a reconstruction matrix of 512 × 512 pixels, slice thickness of 0.5 mm, reconstruction interval of 0.3 mm and convolution kernel FC63. The DICOM series were visualized in the free software InVesalius version 3.1 (CTI, Sao Paulo, Brazil).

The images were generated in the period from 2017 to 2022 entirely for diagnostic purposes. The CT scans were selected by experienced neuroradiologists (D.Z., N.F.) in accordance with the aim of this study. The exclusion criteria during the selection were any traces of trauma, pathological lesions or surgical procedures in the region of the cranial base. The DICOM series were anonymized and only the sex and age of the individuals were used for further statistical analysis. This study was approved by the Human Research Ethics Committee at the Institute of Experimental Morphology, Pathology and Anthropology with Museum, Bulgarian Academy of Sciences. 

The SBs were categorized according to the classification of Archana et al. [7], which included 4 main types of bridges: Type I—a bridge between the anterior and middle clinoid processes; Type II—a bridge between the anterior, middle and posterior clinoid processes; Type III—a bridge between the anterior and posterior clinoid processes; Type IV—a bridge between the middle and posterior clinoid processes. Type I corresponded to CcB, Types II–IV represented IcBs. Depending on the extent of fusion between the osseous bars, these major types were further subdivided into 4 subtypes according the classification of Keyes [5]: complete type—a complete fusion between the bony bars; contact type—a dividing line or suture between the bony bars; incomplete type—a spicule of bone extending from one clinoid process towards the other with a gap in between; and mixed type—a combination of any of the above subtypes present between the adjacent clinoid processes (only in Type II). The PsB and PaB were categorized as complete or incomplete. 

The chi-square test (χ^2^) was applied to assess the bilateral and sex differences in the distribution of the sphenoid bridges. The Spearman’s correlation analysis was applied to evaluate the strength and direction of the correlations between the bilateral and intertype occurrence of the sphenoid bridges. The correlations’ strength was interpreted based on the absolute values of the correlation coefficients: 0.00–0.30 little if any; 0.30–0.50 low; 0.50–0.70 moderate; 0.70–0.90 high; 0.90–1.00 very high [8]. The positive values indicated positive correlations, while the negative ones denoted negative correlations.

## 3. Results

### 3.1. Sellar (Caroticoclinoid and Interclinoid) Bridges

#### 3.1.1. Frequency

**Figure 1:**
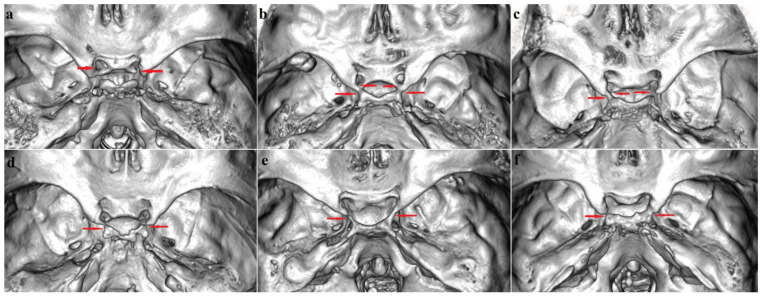
Sellar bridges: (**a**) CcB (Type I): an incomplete CcB on the right side and a complete CcB enclosing CcF on the left side; (**b**) IcB (Type II): a complete form on both sides; (**c**) IcB (Type II) mixed type: an incomplete CcB and IcB on the right side and an incomplete CcB (Type I) on the left side; (**d**) IcB (Type II) mixed type: an incomplete CcB and contact IcB on both sides; (**e**) IcB (Type III): a complete IcB on the right side and an incomplete IcB on the left side; (**f**) IcB (Type III): a complete IcB on the right side and a contact IcB on the left side. Abbreviations: CcB—caroticloclinoid bridge, CcF—caroticoclinoid foramen; IcB—interclinoid bridge. Designations: red arrows point to the osseous bridges (complete and incomplete ones).

The most common sphenoid bridge type was the group of the SBs with a combined overall frequency of 21.7% (20.6% in males and 22.8% in females). The most frequent was CcB-Type I (Figure 1a), followed by IcB-Type II (Figure 1b–d) and IcB-Type III (Figure 1e,f), while IcB-Type IV was not observed at all. The total incidence of CcB (including complete, contact and incomplete types) was 14.3% (11.5% in males and 17.1% in females). The incidence of complete and contact types of CcB enclosing CcF was 9.5% (7.8% in males and 11.1% in females). The frequency of incomplete CcB was 4.8% (3.7% in males and 6.0% in females). The complete and contact types of CcB were 65.9% (67.7% in males and 64.9% in females) of all CcB cases, while the incomplete type was 34.1% (32.3% in males and 35.1% in females).

In the investigated sample the total frequency of IcB-Types II and III was 7.4% (9.1% in males and 5.7% in females). The incidence of complete IcB-Types II and III (including the complete, contact and mixed types) was 5.7% (7.1% in males and 4.5% in females). The incidence of incomplete IcB-Types II and III was 1.6% (2.0% in males and 1.2 in females) (Table 1). The complete and contact forms of IcB Type II represented 40.0% (53.3% in males and 26.7% in females) of all IcB Type II cases; the incomplete IcB Type II was 6.7% (absent in males and in 13.3% of females), and the mixed form was 53.3% (46.7% in males and 60.0% in females). The complete and contact forms of IcB Type III together were equally distributed with the incomplete form of IcB and were found in 50.0% (50.0% in males and 50.0% in females). 

#### 3.1.2. Bilateral and Sex Distribution

The SBs were observed bilaterally in 14.9% (n = 22) of the males. The SBs were observed unilaterally in 11.5% (n = 17) of the cases; in 7.4% (n = 11) of the cases the SBs were found on the right side and in 4.1% (n = 6) on the left side. In the female series, the SBs were observed bilaterally in 18.0% (n = 30) of the cases. In 9.6% (n = 16) the SBs were unilateral; it was right-sided in 6.7% (n = 10) and left-sided in 3.6% (n = 6).

There were no significant bilateral and sex differences in the total frequency of the sellar bridges (combined data for Types I–IV). The distribution pattern of sellar bridge types did not show significant bilateral differences between the sexes. Therefore, data for the right and left sides were pooled for the male and female series and were compared. The comparison showed significant sex differences in the distribution pattern of the sellar bridge types (χ^2^ (4, N = 630) = 8.6, *p* = 0.035). However, the separate comparisons between males and females on the right and left sides did not show any considerable differences. 

### 3.2. Pterygospinous Bridge

#### 3.2.1. Frequency 

**Figure 2:**
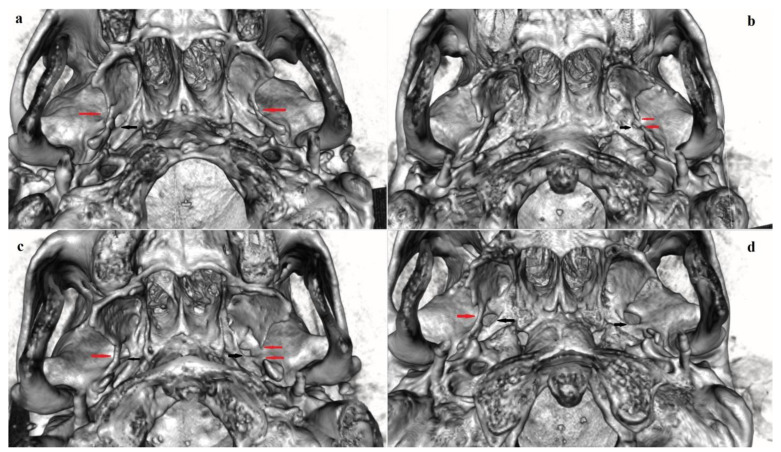
Pterygospinous bridge: (**a**) a bilateral complete PsB, enclosing PsF; (**b**,**c**) a complete PsB on the right side and an incomplete PsB on the left side; (**d**) an unilateral (right-sided) complete PsB. Designations: red arrows point to PsBs; black arrows point to foramen ovale. Abbreviation: PsB—pterygospinous bridge, PsF—pterygospinous foramen.

**Figure 3:**
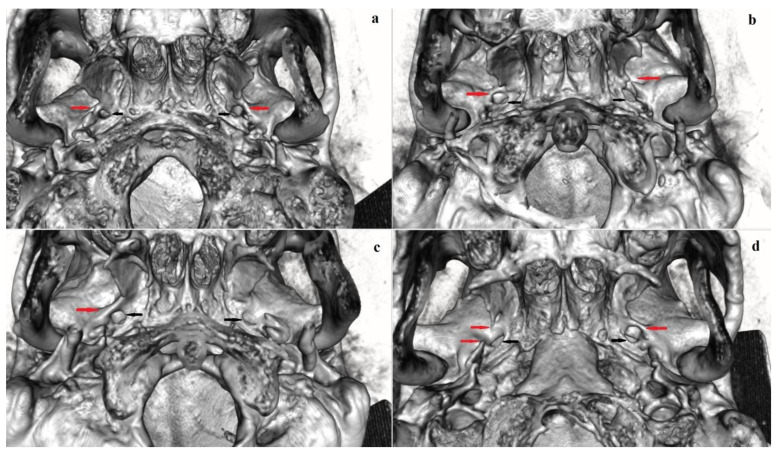
Pterygoalar bridge: (**a**) a bilateral complete PaB, enclosing PaF; (**b**) a complete PaB on the right side and an in complete PaB on the left side; (**c**) a unilateral (right-sided) complete PaB; (**d**) an incomplete PsB on the right side and a complete PaB on the left side. Designations: red arrows point to PsBs; black arrows point to foramen ovale. Abbreviation: PaB—pterygoalar bridge, PaF—pterygoalar foramen, PsB—pterygospinous bridge.

In both sexes and sides, the incomplete PsB was found more frequently compared to the complete bridge forming the PsF (Table 2). The total frequency of PsB (complete and incomplete forms) in the series was 12.4% (16.9% in males and 8.4% in females). A complete PsB (Figure 2) was found in 4.3% of the cases (6.1% in males and 2.7% in females). An incomplete PsB (Figure 2b,c and Figure 3d) was observed in 8.1% (10.8% in males and 5.7% in females). The complete PsB represented 34.6% (36.0% in males and 32.1% in females) of all PsB cases, whereas the incomplete PsB was present in 65.4% (64.0% in males and 67.9% in females).

#### 3.2.2. Bilateral and Sex Distribution

The PsB was bilaterally observed in 8.8% (n = 13) of the males. The unilateral cases of PsB were observed in 16.2% (n = 24); 5.4% (n = 8) right-sided and 10.8% (n = 16) left-sided. In the female series, the PsB was found bilaterally in 5.4% (n = 9) and unilaterally in 6.0% (n = 10), being equally frequent on the right (n = 5; 3.0%) and left side (n = 5; 3.0%).

The total PsB frequency (complete plus incomplete forms) did not show significant bilateral differences in the distribution. Sex differences in the total PsB frequency were significant only concerning the left-side comparison (χ^2^ (1, N = 315) = 8.367, *p* = 0.004). There were no bilateral differences in the distribution pattern of the PsB types (complete and incomplete) in the male and female series. The data for the right and left sides were pooled together and used for comparison of the PsB distribution pattern between males and females. The comparison showed significant differences in the PsB distribution between the sexes (χ^2^ (2, N = 630) = 10.6, *p* = 0.013). The comparison between males and females by sides showed that there was a significant difference in the PsB distribution for the left side (χ^2^ (2, N = 315) = 8.6, *p* = 0.014).

### 3.3. Pterygoalar Bridge

#### 3.3.1. Frequency

The PaB (Figure 3) was found with the lowest frequency of all sphenoid bridges (Table 3). The total frequency of PaB (complete and incomplete forms) was 2.4% (3.0% in males and 1.8% in females). A complete PaB was found in 1.4% of the cases (1.6% in males and 1.2% in females) and an incomplete PaB was found in 1.0% (1.4% in males and 0.6% in females). The complete PaB accounted for 60.0% (55.6% in males and 66.7% in females) of all-PaB cases, whereas the incomplete PaB accounted for 40.0% (44.4% in males and 33.3% in females).

#### 3.3.2. Bilateral and Sex Distribution

The cases of bilateral PaB were found in 1.4 (n = 2) of the male individuals. The PaB was observed unilaterally in 3.4% (n = 5) of the cases; in 2.0% (n = 3) it was on the right side and in 1.4% (n = 2) it was on the left side. The PaB was observed bilaterally only in one case (n = 1; 0.6%) among the female series. The PaB was found unilaterally in 2.4% (n = 4) of the cases; in 1.8% (n = 3) of the cases on the right side and in 0.6% (n = 1) on the left side.

There were no significant bilateral or sex differences in the total PaB distribution or by types (complete and incomplete forms). 

### 3.4. Correlation Analysis

There were no significant correlations between the different types of sphenoid bridges. Each type of bridging showed significant correlations between the right and left side co-occurrence in males and females (Table 4 and Table 5).

### 3.5. Sphenoid Bridges Co-Occurrence

More than one type of bridging in males was found in a total of 11 (7.4%) cases: IcB and PsB (n = 6; 4.1%); CcB and PsB (n = 3; 2.0%); PsB and PaB (n = 1; 0.7%); CcB and IcB and PsB (n = 1; 0.7%). In females, more than one type of bridge was found in a total of 4 (2.4%) cases: IcB and PsB (n = 2; 1.2%); CcB and PsB (n = 1; 0.6%); CcB and PaB (n = 1; 0.6%).

## 4. Discussion

### 4.1. Frequency and Distribution

#### 4.1.1. Sellar (Caroticoclinoid and Interclinoid) Bridges

##### Caroticoclinoid Bridge (Type I)

The total CcB frequency, including complete and incomplete forms, has been reported to vary in different population groups and countries: India—12.00% [7], 16.4% [9] and 22.2% [10]; South Korea—15.7% [11]; Japan—16.6% [12]; Bosnia and Herzegovina—16.8% [13]; UK—17.5% including mixed type 2.5% [14]; Nepal—20% [15]; Turkey—34.2% [16], 35.3% [17] and 35.7% [18]; USA—34.8% [5]; and Greece—60.16% [19] and 74% [20]. Our results show that the total CcB frequency in contemporary Bulgarians is 14.3% (11.5% in males and 17.1% in females), i.e., the CcB in Bulgarians is observed more infrequently than in most of the studied populations.

A complete CcB enclosing the CcF, has been reported to occur with varying frequency in different populations: India—3.0% [9] and 5.2% [7]; South Korea—4.1% [11]; Bosnia and Herzegovina—7.0% [13]; Nepal—8.6% [15]; Italy 8.7% [21]; Turkey—8.8% [18] and 15.0% [22]; UK—10.0% [14]; Poland—16.3% [23]; USA—20% [1], 31.0% [2] and 41.8% [24]; and Greece—20.4% [20] and 23.6% [19]. A previous investigation of Bulgarian males from the beginning of the 20th century [25] has reported a frequency of 8.2% (9 out of 110 dry skulls) for the CcF. In our study, the frequency of complete and contact types of CcB enclosing the CcF is 9.5% (7.8% in males and 11.1% in females), which is within the range of the reported frequencies in the other populations.

Incidences of incomplete CcB have been observed as follows: UK—5.0% [14]; India—6.8% [7] and 13.4% [9]; Bosnia and Herzegovina—9.8% [13]; Turkey—10.0% [22], 14.9% [18]; Nepal—11.4% [15]; South Korea—11.6% [11]; Greece—36.6% [19] and 46.0% [20]; and USA—42.0% [2]. In our study the frequency of incomplete CcB is 4.8% (3.7% in males and 6.0% in females), i.e., below the frequencies reported for the other population groups.

##### Interclinoid Bridges (Types II, III and IV)

Total IcB, including complete and incomplete forms, has been observed with varying frequency in different population groups: Japan—2.8% [12]; India—4.0% [7] and 6.7% [9]; USA—8.7% [5]; UK—22.1% including mixed type 1.3% [14]; Poland—11.8% [26]; Turkey—11.8% [17], 34.2% [16]; and Greece—22.0% [19]. Our results show that IcB frequency (Types II and III) is 7.4% (9.1% in males and 5.7% in females), which is commensurable with the frequency in the other populations.

Complete IcB has been found to vary among different groups: India—0.9% [10], 1.5% [9] and 1.6% [7]; Turkey—5.0% [22], 5.9% [17], 6.0% [27] and 8.2% [18]; UK—5.4% [14]; USA—8.2% [28]; Italy—13.0% [29] and 16.00% [21]; and Poland—13.8% [23]. Previous investigation of Bulgarian males from the beginning of the 20th century [25] has reported an IcB frequency of 7.3% (8 out of 110 dry skulls; in 4 cases it was bilateral and in 4 cases unilateral: 3 on the right side and 1 on the left side; Type II). In our study, the incidence of complete IcB (Type II and III) is slightly lower: 5.7% (7.1% in males and 4.5% in females), including the complete, contact and mixed type.

Incidences of incomplete IcB vary in different geographic regions and have been reported as follows: India—2.4% [7] and 5.2% [9]; Turkey—5.9% [17]; UK—15.4% [14]; Italy—30.0% [29]; and USA—38.4% [28]. In our study, the incidence of incomplete IcB (Types II and III) is 1.6% (2.0% in males and 1.2 in females), which is lower than the other reported frequencies.

In our study the combined overall frequency of SBs (CcB and IcB) is 21.7%. The most common is CcB (Type I), formed between the anterior and middle clinoid process. Less common is the bony fusion between the anterior and posterior clinoid processes (Type III), while Type IV is not observed at all. 

#### 4.1.2. Pterygospinous Bridge

The total PsB frequency, including complete and incomplete bridges, has been reported to vary in the different population groups: USA—1.3% [30]; India—8.8% [31], 9.6% [32], 9.7% [9], 10.2% [33], 17.0% [34] and 17.3% [35]; UK—16.1% [36], 17.1% (including mixed type 2.5%) [14]; South Korea—18.0% [37]; Greece—27.0% [38]; and Brazil—28.0% [39]. Our study shows a total frequency of PsB of 12.4% (16.9% in males and 8.4% in females). 

The reported incidences of complete PsB among different populations vary in wide ranges: USA—0.7% [30], 5.5% [40], 6.3 % [3]; India—1.0% [34], 2.7% [35], 3.0% [9], 3.9% [41], 4.0% [33], 5.7% [32] and 7.5% [31]; South Korea—1.4% [37]; Greece—2.0% [38]; UK—2.1% [14], 4.4% [36]; Poland 5.0% [42]; Turkey—5.5% (fixed cadavers) and 8.8% (dry skulls) [16]; Japan and Germany—6.0% [4]; and Brazil 8.6% [39]. In a previous investigation of Bulgarian males, PsF has been found in 5.6% (151 out of 2709 crania) [25]. In 35.1% (n = 53) of the cases the PsF has been bilateral; PsF has been right-sided in 26.5% (n = 40) of the crania and left-sided in 38.4% (n = 58) of the crania [25]. According to the present study, complete PsB in Bulgarians occurs in 4.3% (6.1% in males and 2.7% in females). An extensive meta-analysis combining 35 separate studies (n = 14,047 subjects) has investigated the total frequency, morphologic and morphometric characteristics of PsB, and its probable racial and gender differences among populations [43]. According to the investigation, the overall pooled frequency of complete PsB is 4.4%. A complete PsB has been found most often in Europe with a pooled prevalence of 4.9%, followed by South (4.5%) and North America (4.4%), but these differences are not significant. The sex distribution has shown that the complete PsB is considerably more frequent in males (5.7%) than in females (2.4%). The PsB has been more commonly found unilaterally (23.7%) compared to the bilateral cases (8.0%), although the differences are insignificant. Complete PsB has been observed more frequently on the left side (53.1%) in comparison to the right-side occurrence (46.9%). According to our results, the complete PsB is more commonly found in males than in females and on the left side compared to the right one. An incomplete PsB has been observed with varying frequency in different groups: USA—0.7% [30] and 28.71% [40]; India—1.0% [44], 1.3% [31], 3.01% [45], 3.9% [32], 6.2% [33] 6.7% [9], 14.7% [35], 16.0% [34] and 33.0% [41]; UK—11.7% [36] and 12.5% [14]; South Korea—16.6% [37]; Brazil—19.4% [39]; and Greece—25.0% [38]. Incomplete PsB in Bulgarians occurs in 8.1% (10.8% in males and 5.7% in females). The frequency of incomplete PsB has been studied, combining data from 28 different studies (n = 9124 subjects) [43]. The incomplete PsB frequency (11.6%) has been significantly higher compared to that of the complete PsB (4.4%). Geographical location has shown variable occurrences of incomplete PsB. The meta-analysis revealed that the incomplete PsB has been most prevalent among Europeans (15.4%), followed by South Americans (15.3%) and North Americans (12.6%), with the lowest pooled frequency found in Asians (8.4%); though, the differences are insignificant. No significant sex differences in the incomplete PsB distribution have been found. The incomplete PsB has been observed more often unilaterally (45.3%; 50.8% on the left side and 49.2% on the right side) than bilaterally (19.3%) [43]. In our study the incomplete PsB is more frequently observed in males than in females; the PsB is more common on the left side in males and on the right side in females.

#### 4.1.3. Pterygoalar Bridge

The total PaB frequency, including complete and incomplete forms, has been reported to vary in different populations: USA—1.3% [30]; UK—6.3% including mixed type 0.8% [14]; Greece—8.0% [38] and 31.7% [46]; South Korea—8.4% [37]; India—30.0% [34]; and Brazil—62.4% [39]. In an antecedent study of Bulgarian males, the PaF has been found in 149 (5.5%) out of 2709 investigated crania. The PaF has been observed bilaterally in 101 (67.8%) of crania; it has been more rarely recorded unilaterally and almost equally distributed on the right and left sides [25]. Our study shows a total PaB frequency of only 2.4% (3.0% in males and 1.8% in females).

Incidence of complete PaB among different groups has been found as follows: UK—0.7% [36] and 1.3% [14]; USA—0.7% [30], 5.9% [40] and 10.3% [3]; India—1.0% [34]; South Korea—2.8% [37]; Greece—4.1% [46] and 7.0% [38]; Turkey—4.9% (fixed cadavers) and 7.9% (dry skulls) [16]; and Brazil—12.9% [39]. According to our study, complete PaB in Bulgarians occurs in 1.4% of the cases (1.6% in males and 1.2% in females). The combined data of 25 articles (n = 16,168 subjects) have been assessed in a meta-analysis considering the complete PaB frequency, which is found to be 4.4% [47]. The geographical distribution has shown that complete PaB is most commonly found in Asia (7.0%) followed by South and North Americas (5.0%), and Europe (2.6%), but these differences are statistically insignificant. Complete PaB has been observed more commonly in males (6.7%) than in females (4.5%), but this difference is not considerable. Complete PaB has been most frequently recorded unilaterally (36.6%), on the left side (53.2%). Bilateral PaB has been statistically less common, with a pooled frequency of 5.0% [47].

Incidences of incomplete PaB have been found as follows: USA—0.7% [30] and 17.8% [40]; Greece—1.0% [38] and 27.60% [46]; UK—4.2% [14]; South Korea—5.6% [37]; India—29.0% [34]; and Brazil—49.4% [39]. Incomplete PaB in Bulgarians occurs in 1.0% (1.4% in males and 0.6% in females). The incomplete PaB frequency (8.4%) has been studied in a meta-analysis combining 19 studies (n = 6404 subjects) [47]. Geographic distribution has shown that incomplete PaB is most commonly observed in South Americans (19.2%), followed by Asians (7.0%), Europeans (6.0%) and North Americans (5.6%), although the differences are not significant. The incomplete PaB has been observed with a pooled frequency of 18.6% among males and 15.6% among females, but there are no significant sex differences in its distribution. Unilateral incomplete PaB (43.8%), present on the left side (52.0%), has been more frequent than the bilaterally presented incomplete PaB (12.5%) [47]. 

#### 4.1.4. Sphenoid Bridges Co-Occurrence and Correlation Analysis

We did not find any significant correlations in the occurrence of the different sphenoid bridge types, similarly to the observation of Ossenberg [48]. However, each type of bridging showed significant correlations between the right and left side occurrence in males and females. 

Ps and Pa foramina may be presented together in one individual on the same side [6]. However, there are no reports about the distribution of the mandibular nerve branches in a case of Ps and Pa bridges coexistence. Such a case has not been documented among our series either. 

### 4.2. Etiology

It has been accepted that the osseous bridge between the anterior and middle clinoid processes arises through ossification of the caroticoclinoid ligament or a dural fold stretched between them [49]. A bone connection between the posterior, middle and anterior clinoid processes, the posterior and anterior processes or, rarely, between the posterior and middle processes, the so-called sellar bridges (interclinoid taenia), has been thought to be a result of ossification of the interclinoid ligament, which connects the clinoids [5]. The Ps and Pa bridges have been attributed to a secondary senile ossification of Ps (Civinini’s) and Pa (Hyrtl’s) ligaments, respectively [3]. Ps and Pa ligaments are in close proximity, but they are distinct in their courses, most notably posteriorly, with the Ps ligament (a thickening of the interpterygoid aponeurosis) attaching to the spine of the sphenoid and the Pa ligament (a thickening of the lateral interpterygoid or pterygotemporomaxillary aponeurosis) attaching more laterally to the undersurface of the sphenoid [6]. 

Ligaments are dense, fibrous connective tissue that attach skeletal elements to each other and transmit mechanical forces [50]. They are filamentous collagen structures, of which the extracellular matrix is the major part and consist mainly of collagen, and specifically collagen Type I [51]. The cell populations in ligaments include fibroblasts embedded between parallel chains of collagen fibrils and small subsets of progenitor cells. Under normal conditions, ligamentocytes are thought to be quiescent and have very low rates of proliferation, while extracellular matrix production is responsive to changes in the mechanical load. Cells in ligaments similar to those in tendons respond to mechanical strain by increasing collagen production, and mechanical signals promote mesenchymal stem cell differentiation into ligamentocytes. Mechanical stimulation also promotes collagen fiber thickening along with increased fiber density [50]. Mineralization of ligaments is commonly recognized as an age-dependent phenomenon. Age-related changes in ligaments are associated with a significant increase in mineral content (Ca and P) and decrease in all extracellular matrix components (elastin, elastin cross-links, fibrillin, collagen and glycoprotein) [52]. Ossification of ligaments involves multiple etiological factors, including genetic factors, dietary habits, metabolic abnormalities, and some local factors such as mechanical stress, which could induce mineralization [53]. The pathophysiology of heterotopic/ectopic ligament ossification is still unclear, but it is mostly believed that it is a failed injury repair process. The etiology of heterotopic ligament ossification is generally considered a tissue repair process similar to fracture repair, which is a dynamic, highly complex and arranged physiological process and includes trauma/injury, inflammation, mesenchymal stromal cell recruitment, chondrogenic differentiation and ossification formation [51]. 

The exact factors which could possibly induce mineralization of the skull base ligaments are still not fully understood. Chewing on one side has been considered as a factor responsible for the Ps and Pa bridge formation through development of bony bars in the fibers of the pterygoid muscles. However, their position would be expected to be in the muscle tendon rather than between the two parts of sphenoid bone [3]. Pathological bone formation as a response to an infection has also been considered as a possible factor for the Ps and Pa bridge formation; though, the smooth contours of the bars and the lack of other accompanying bone lesions are atypical for such a process [3]. It has also been suggested that the CcB and IcB are laid down in cartilage at an early stage of development (prenatal life) and ossify in early childhood [54] (Lang, 2001). The reported cases of sellar osseous bridges in fetuses, infants [5,49] and young children [14,55], and the observed lack of significant age-dependent differences in the Ps and Pa bridges’ occurrence [3,40] as well as their presence in young children [36,56] have been considered evidence that these structures are developmental abnormalities arising during the formation of the chondrocranium [49,55,57], rather than a secondary ossification of ligaments induced by mechanical stress or inflammation. Thus, a genetic background has been proposed [36,43], but the exact etiology of the sphenoid osseous bridge formation remains largely unknown.

Ps (Civinini’s) ligament has been considered a local reinforcement of the superior part of the interpterygoid fascia [4]. It could be short, fastened higher up on the plate, or two ligaments (long and short) may be presented [58]. Besides the Ps ligament and osseous bridge, there could also be found a Ps muscle [6], which inserts into the area between the sphenoidal spine and the fissura petrotympanica or into the temporomandibular joint capsule reaching the articular disc [4]. The existence of these anatomical peculiarities is well-known. They have been described as osseous, fibrous, or muscular in character and have been considered one and the same structure, which may change its character during the course of life. However, many arguments including their simultaneous co-occurrence support the hypothesis that each of these structures possesses its own identity [4]. This also suggests that the PsB is not simply a secondary ossified Ps ligament.

### 4.3. A Phylogenetic Aspect of the Development and Relationship between the Spinous Process, Processus Angularis and the Prominence of the Lateral Pterygoid Plate 

Processus angularis, in the sense of a backwardly directed postero-lateral prolongation of the alisphenoid inserted between the squamous and petrous portions of the temporal, is a common mammalian possession; spina angularis (spinous process; sphenoidal spine), in the sense of an independent spine of bone developed on the processus angularis, is a peculiar human development [59]. In lemurs processus angularis is prolonged backwards, the spinous process is not developed, and the prominence of the lateral pterygoid plate passes medial to the region of the processus angularis. In tarsiers the spinous process is not developed as well. In platyrrhines the spine also fails to develop, and the posterior extremity of the lateral pterygoid plate falls short of or passes medial to the region of the processus angularis. In Old World monkeys the lateral pterygoid plate is prolonged to the apex of the processus angularis on which a prominent ridge is presented. This ridge passes lateral to the foramen ovale and constitutes a complete PsB. In orangutans the processus angularis is reduced and there is no prominence corresponding to a spinous process; the prominence of the lateral pterygoid plate either ends before reaching the angular area or else passes to it medial to the foramen ovale. In chimpanzees processus angularis is reduced and in gorillas it is practically absent, being represented only by a spicule of bone posterior (not lateral) to the foramen ovale. In African anthropoids the lateral pterygoid plate passes medial to the foramen ovale, the processus angularis is reduced and there is no spinous process. However, in gorillas, there is a bony prominence on the temporal squama called spina angularis temporalis [59]. 

In general, a caudal extension of the lateral sphenoid lamina in lemurs passes medial to the foramen ovale and transmits part of the third division of the trigeminal nerve towards the medial side. In pithecoid condition, the PsB is complete and passes lateral to the foramen ovale transmitting the nerve to the lateral side. In Man and Anthropoids, the PsB is usually incomplete. In cases when the PsB is connected to the sphenoid spine, it could pass laterally to the foramen ovale (pithecoid), medially (primitive) or across the lumen of the foramen [59]. The presence of PsB and PaB has been considered atavistic in nature since the PaB is more or less generally present in higher apes [3]. Furthermore, the existence of a wide PsB has been noted in skulls of herbivora, rodentia, carnivora, and Old World monkeys, but never in skulls of New World monkeys. Thus, the PsB in humans has been considered a phylogenetic remnant [4].

### 4.4. Clinical Significance

Osseous bridges of the sphenoid bone form additional foramina, which most commonly facilitate the passage of nerves and vessels through the cranial base. However, these bridges could entrap and compress the passing neurovascular structures causing lesions as a result of pressure or mechanical irritation [25]. Furthermore, such a foramen completed in infancy could be narrowed with ageing by a progressive deposition of bone at the margins, which could also result in compression of any structures traversing it [36]. Finally, replacement of cranial base ligaments with osseous bridges obstructs surgical corridors exerting a significant impact on regional neurosurgery [1,2,4]. 

#### 4.4.1. Sellar (Caroticoclinoid and Interclinoid) Bridges

Basically, the clinical significance of SBs is related to their proximity to the clinoid segment of the ICA and cavernous sinus. SBs could potentially compress these structures giving rise to various clinical symptoms [7]. In particular, the CcB could cause an ICA compression, tightening or stretching, which may result in an insufficient blood supply to the brain [27]. The CcB and IcB complicate the removal of the anterior clinoid process (anterior clinoidectomy) and increase the risk of carotid laceration [2]. Anterior clinoidectomy is necessary to expose the cavernous sinus and to access the ICA clinoid segment for management of aneurysms, neoplasms and traumatic lesions in the parasellar region [7,14]. Moreover, the middle clinoid process is a reliable landmark for localization of the anteromedial roof of the cavernous sinus and the transition between the intracavernous and paraclinoidal segments of the ICA during endoscopic endonasal approach to the sellar/parasellar/suprasellar region [1]. Involvement of the middle clinoid process in the formation of a CcF complicates the middle clinoidectomy, which is helpful in managing sellar tumors with true invasion of the cavernous sinus through an endonasal approach extended to the parasellar region [1,2]. 

#### 4.4.2. Pterygospinous and Pterygoalar Bridges

From a clinical perspective, the infratemporal fossa is a region of the skull base, which is difficult for surgical access, and the presence of partial or complete osseous bridges additionally obstructs it. This particularly concerns the trans-zygomatic approach to the external skull base and the superior part of the para- and retropharyngeal space, in which the pterygoid process and the spine of the sphenoid bone are well-known landmarks [4,6]. The Ps and Pa bridges occupy the high and deep portion of the infratemporal fossa near the foramen ovale, which makes them of significant importance in anesthetic treatment of the mandibular nerve and therapeutics of the trigeminal ganglion [60]. The Ps and Pa bridges interact with the mandibular nerve and its branches and could potentially cause entrapment neuropathies and occlusion of vessels [36]. Compression or entrapment of motor branches of the mandibular nerve could lead to paresis or weakness in the innervated muscle, and that of sensory branches can provoke neuralgia or paraesthesia [34,46]. The osseous bridges below the foramen ovale could also cause an artery angulation resulting in a restriction of blood flow and possible ischemia of some neurons in the trigeminal ganglion [36]. 

Particularly, the PsB could entrap the stem or branches of the mandibular nerve [4]. In cases of an enlarged lateral pterygoid plate or PsB, the course of the lingual and the inferior alveolar nerves shifts in a way that the inferior alveolar nerve could be entrapped between the bone and the pterygoid muscles, which increases the risk of compression and mandibular neuralgia [42]. An entrapment of the lingual nerve between the PsB and the medial pterygoid muscle has been observed [61,62]. Such an entrapment could cause numbness in the supplied regions; i.e., the mucosa of the floor of the mouth, the presulcal part of the tongue, and the lingual gingiva [61]. The PsB has also been reported to divide the lingual nerve fibers into anterior and posterior parts. Thus, the anterior fibers lie between the tensor veli palatini muscle and PsB, making it vulnerable to compression [60]. It has been suggested that PsB may potentially compress the auriculotemporal branch, causing periauricular sensory or parotid glandular secretomotor symptoms [14,30]. The chorda tympani, a branch of the facial nerve, could also be compressed by the PsB which may result in an impaired taste sensation from the anterior two thirds of tongue [44]. Furthermore, the PsB could occlude the blood vessels supplying the trigeminal ganglion [36].

The PaB could potentially compress the deep temporal, lateral pterygoid and masseteric nerve, which could lead to paresis or weakness in the innervated mastication muscles and may cause chewing disorders [38]. Compression of the buccal nerve, a sensory branch deriving from the anterior trunk of the mandibular nerve, can result in neuralgia-like paroxysmal pain or numbness of the buccal region [46]. Due to its position, the PaB acts as a mechanical barrier and complicates the passage of a needle in percutaneous transoval procedures for treating complex trigeminal neuralgias, trigeminal rhizotomy or cavernous sinus biopsy [3,30,63]. 

## 5. Conclusions

The presence of osseous bridges instead of the ligamentous connection between separate parts of the sphenoid bone is a relatively common finding in the investigated series. The SBs are the most common type of sphenoid bridging; particularly, the CcB, followed by the PsB and the PaB. The total SBs frequency did not show significant bilateral or sex differences. However, there were considerable sex differences in the SB distribution by types. The PsB did not show significant bilateral differences but showed considerable sex differences concerning the left-side frequency, which was significantly higher in the male series. The PaB did not show any considerable bilateral or sex differences in its distribution. The correlation analysis did not indicate significant relationships between the different types of sphenoid bone bridges. However, each type of bridging showed significant positive correlations between the right and left side co-occurrence in males and females.

## Figures and Tables

**Table 1:** Frequency of sellar bridges by types in both sides and sexes.

Sellar Bridges	Males						Females					
	Right		Left		R + L		Right		Left		R + L	
	n	%	n	%	n	%	n	%	n	%	n	%
Type 1	19	12.8	15	10.1	34	11.5	31	18.6	26	15.6	57	17.1
complete	12	8.1	9	6.1	21	7.1	15	9.0	15	9.0	30	9.0
contact	2	1.3	0	0.0	2	0.7	4	2.4	3	1.8	7	2.1
incomplete	5	3.4	6	4.0	11	3.7	12	7.2	8	4.8	20	6.0
Type 2	9	6.1	6	4.0	15	5.1	8	4.8	7	4.2	15	4.5
complete	3	2.0	4	2.7	7	2.4	2	1.2	2	1.2	4	1.2
contact	1	0.7	0	0.0	1	0.3	0	0.0	0	0.0	0	0.0
incomplete	0	0.0	0	0.0	0	0.0	2	1.2	0	0.0	2	0.6
mixed	5	3.4	2	1.3	7	2.4	4	2.4	5	3.0	9	2.7
Type 3	5	3.4	7	4.7	12	4.0	1	0.6	3	1.8	4	1.2
complete	4	2.7	1	0.7	5	1.7	1	0.6	0	0.0	1	0.3
contact	1	0.7	0	0.0	1	0.3	0	0.0	1	0.6	1	0.3
incomplete	0	0.0	6	4.0	6	2.0	0	0.0	2	1.2	2	0.6
Type 4	0	0.0	0	0.0	0	0.0	0	0.0	0	0.0	0	0.0
Types 1–4	33	22.3	28	18.9	61	20.6	40	24.0	36	21.6	76	22.8
Absence	115	77.7	120	81.1	235	79.4	127	76.0	131	78.4	258	77.2
Total	148	100.0	148	100.0	296	100.0	167	100.0	167	100.0	334	100.0

**Table 2:** Frequency of pterygospinous bridge in both sides and sexes.

PsB/Types	Males						Females					
	Right		Left		R + L		Right		Left		R + L	
	n	%	n	%	n	%	n	%	n	%	n	%
PsB	21	14.2	29	19.6	50	16.9	14	8.4	14	8.4	28	8.4
complete	8	5.4	10	6.8	18	6.1	3	1.8	6	3.6	9	2.7
incomplete	13	8.8	19	12.8	32	10.8	11	6.6	8	4.8	19	5.7
Absence	127	85.8	119	80.4	246	83.1	153	91.6	153	91.6	306	91.6
Total	148	100.0	148	100.0	296	100.0	167	100.0	167	100.0	334	100.0

**Table 3:** Frequency of pterygoalar bridge in both sides and sexes.

PaB/Types	Males						Females					
	Right		Left		R + L		Right		Left		R + L	
	n	%	n	%	n	%	n	%	n	%	n	%
PaB	5	3.4	4	2.8	9	3.0	4	2.4	2	1.2	6	1.8
complete	3	2.0	2	1.4	5	1.6	3	1.8	1	0.6	4	1.2
incomplete	2	1.4	2	1.4	4	1.4	1	0.6	1	0.6	2	0.6
Absence	143	96.6	144	97.2	287	97.0	163	97.6	165	98.8	328	98.2
Total	148	100.0	148	100.0	296	100.0	167	100.0	167	100.0	334	100.0

**Table 4:** Correlation between the different types of sphenoid bone bridges in the male series.

Males								
			SB-R	SB-L	PsB-R	PsB-L	PaB-R	PaB-L
Spearman’s rho	SB-R	Correlation Coefficient	1.000	0.653	−0.078	−0.019	−0.100	−0.089
		Sig. (2-tailed)	.	0.000	0.344	0.818	0.226	0.281
	SB-L	Correlation Coefficient		1.000	−0.048	0.022	−0.090	−0.081
		Sig. (2-tailed)			0.562	0.788	0.275	0.331
	PsB-R	Correlation Coefficient			1.000	0.433	−0.076	0.052
		Sig. (2-tailed)			.	0.000	0.358	0.533
	PsB-L	Correlation Coefficient				1.000	−0.092	−0.082
		Sig. (2-tailed)					0.264	0.320
	PaB-R	Correlation Coefficient					1.000	0.430
		Sig. (2-tailed)					.	0.000
	PaB-L	Correlation Coefficient						1.000
		Sig. (2-tailed)						.

SB-R—sellar bridge on the right side; SB-L—sellar bridge on the left side; PsB-R—pterygospinous bridge on the right side; PsB-L—pterygospinous bridge on the left side; PaB-R—pterygoalar bridge on the right side; PaB-L—pterygoalar bridge on the left side.

**Table 5:** Correlation between the different types of sphenoid bone bridges in the female series.

Females								
			SB-R	SB-L	PsB-R	PsB-L	PaB-R	PaB-L
Spearman’s rho	SB-R	Correlation Coefficient	1.000	0.729	−0.069	−0.069	−0.088	0.067
		Sig. (2-tailed)	.	0.000	0.379	0.379	0.259	0.388
	SB-L	Correlation Coefficient		1.000	−0.001	−0.001	−0.082	0.076
		Sig. (2-tailed)		.	0.990	0.990	0.291	0.328
	PsB-R	Correlation Coefficient			1.000	0.610	−0.047	−0.033
		Sig. (2-tailed)			.	0.000	0.543	0.669
	PsB-L	Correlation Coefficient				1.000	−0.047	−0.033
		Sig. (2-tailed)				.	0.543	0.669
	PaB-R	Correlation Coefficient					1.000	0.343
		Sig. (2-tailed)					.	0.000
	PaB-L	Correlation Coefficient						1.000
		Sig. (2-tailed)						.

SB-R—sellar bridge on the right side; SB-L—sellar bridge on the left side; PsB-R—pterygospinous bridge on the right side; PsB-L—pterygospinous bridge on the left side; PaB-R—pterygoalar bridge on the right side; PaB-L—pterygoalar bridge on the left side.

## Data Availability

The data presented in this study are available upon request from the corresponding author.

## References

[1] Fernandez-Miranda J.C., Tormenti M., Latorre F., Gardner P., Snyderman C. (2012). Endoscopic endonasal middle clinoidectomy. Oper. Neurosurg..

[2] Sharma A., Rieth G.E., Tanenbaum J.E., Williams J.S., Ota N., Chakravarthi S., Manjila S., Kassam A., Yapicilar B. (2018). A morphometric survey of the parasellar region in more than 2700 skulls: Emphasis on the middle clinoid process variants and implications in endoscopic and microsurgical approaches. J. Neurosurg..

[3] Chouké K.S. (1946). On the incidence of the foramen of Civinini and the Porus crotaphitico-buccinatorius in American whites and negroes. Am. J. Physiol. Anthropol..

[4] Von Lüdinghausen M., Kageyama I., Miura M., Alkhatib M. (2006). Morphological peculiarities of the deep infratemporal fossa in advanced age. Surg. Radiol. Anat..

[5] Keyes J.E.L. (1935). Observations on four thousand optic foramina in human skulls of known origin. Arch. Ophthalmol..

[6] Lepp F.H., Sandner M.O. (1968). Anatomic-radiographic study of ossified pterygospinous and “innominate” ligaments. Oral Surg. Oral Med. Oral Pathol..

[7] Archana R., Anita R., Jyoti C., Punita M., Rakesh D. (2010). Incidence of osseous interclinoid bars in Indian population. Surg. Radiol. Anat..

[8] Hinkle D.E., Wiersma W., Jurs S.G. (2003). Applied Statistics for the Behavioral Sciences.

[9] Aggarwal B., Gupta M., Kumar H. (2012). Ossified ligaments of the skull. J. Anat. Soc. India.

[10] Suprasanna K., Kumar A. (2017). Surgically relevant bony anatomical variations in paraclinoid aneurysms-three-dimensional multi-detector row computed tomography-based study. J. Neurosci. Rural Pract..

[11] Lee H.Y., Chung I.H., Choi B.Y., Lee K.S. (1997). Anterior clinoid process and optic strut in Koreans. Yonsei Med. J..

[12] Ota N., Tanikawa R., Miyazaki T., Miyata S., Oda J., Noda K., Tsuboi T., Takeda R., Kamiyama H., Tokuda S. (2015). Surgical microanatomy of the anterior clinoid process for paraclinoid aneurysm surgery and efficient modification of extradural anterior clinoidectomy. World Neurosurg..

[13] Kapur E., Mehić A. (2012). Anatomical variations and morphometric study of the optic strut and the anterior clinoid process. Bosn. J. Basic Med. Sci..

[14] Touska P., Hasso S., Oztek A., Chinaka F., Connor S.E.J. (2019). Skull base ligamentous mineralisation: Evaluation using computed tomography and a review of the clinical relevance. Insights Imaging.

[15] Gupta N., Ray B., Ghosh S. (2005). A study on anterior clinoid process and optic strut with emphasis on variations of caroticoclinoid foramen. Nepal Med. Coll. J..

[16] Peker T., Karaköse M., Anil A., Turgut H.B., Gülekon N. (2002). The incidence of basal sphenoid bony bridges in dried crania and cadavers: Their anthropological and clinical relevance. Eur. J. Morphol..

[17] Boyan N., Ozsahin E., Kizilkanat E., Tekdemir I., Soames R., Oguz O. (2011). Surgical importance of the morphometry of the anterior clinoid process, optic strut, caroticoclinoid foramen, and interclinoid osseous bridge. Neurosurg. Q..

[18] Erturk M., Kayalioglu G., Govsa F. (2004). Anatomy of the clinoidal region with special emphasis on the caroticoclinoid foramen and interclinoid osseous bridge in a recent Turkish population. Neurosurg. Rev..

[19] Natsis K., Piagkou M., Lazaridis N., Totlis T., Anastasopoulos N., Constantinidis J. (2018). Incidence and morphometry of sellar bridges and related foramina in dry skulls: Their significance in middle cranial fossa surgery. J. Cranio Maxillofac. Surg..

[20] Efthymiou E., Thanopoulou V., Kozompoli D., Kanellopoulou V., Fratzoglou M., Mytilinaios D., Piagkou M., Johnson E.O. (2018). Incidence and morphometry of caroticoclinoid foramina in Greek dry human skulls. Acta Neurochir..

[21] Gibelli D., Cellina M., Gibelli S., Panzeri M., Oliva A.G., Termine G., Sforza C. (2018). Sella turcica bridging and ossified carotico-clinoid ligament: Correlation with sex and age. Neuroradiol. J..

[22] Dagtekin A., Avci E., Uzmansel D., Kurtoglu Z., Kara E., Uluc K., Akture E., Baskaya M.K. (2014). Regional microsurgical anatomy and variations of the anterior clinoid process. Turk. Neurosurg..

[23] Skrzat J., Mroz I., Marchewka J. (2012). Bridges of the sella turcica–anatomy and topography. Folia Med. Crac..

[24] Miller C., Chamoun R., Beahm D. (2016). Morphometric analysis of the middle clinoid process using maxillofacial computed tomography scans. Oper. Neurosurg..

[25] Kadanoff D., Mutafov S. (1984). The Human Skull in a Medico-Anthropological Aspect: Form, Dimensions and Variability.

[26] Kucia A., Jankowski T., Siewniak M., Janiszewska-Olszowska J., Grocholewicz K., Szych Z., Wilk G. (2014). Sella turcica anomalies on lateral cephalometric radiographs of Polish children. Dentomaxillofacial. Radiol..

[27] Özdoğmuş Ö., Saka E., Tulay C., Gürdal E., Uzün I., Cavdar S. (2003). The anatomy of the carotico-clinoid foramen and its relation with the internal carotid artery. Surg. Radiol. Anat..

[28] Cederberg R.A., Benson B.W., Nunn M., English J.D. (2003). Calcification of the interclinoid and petroclinoid ligaments of sella turcica: A radiographic study of the prevalence. Orthod. Craniofacial Res..

[29] Scribante A., Sfondrini M.F., Cassani M., Fraticelli D., Beccari S., Gandini P. (2017). Sella turcica bridging and dental anomalies: Is there an association?. Int. J. Paediatr. Dent..

[30] Tubbs R.S., May W.R., Apaydin N., Shoja M.M., Shokouhi G., Loukas M., Cohen-Gadol A.A. (2009). Ossification of ligaments near the foramen ovale: An anatomic study with potential clinical significance regarding transcutaneous approaches to the skull base. Neurosurgery.

[31] Saran R.S., Ananthi K.S., Subramaniam A., Balaji M.T., Vinaitha D., Vaithianathan G. (2013). Foramen of Civinini: A new anatomical guide for maxillofacial surgeons. J. Clin. Diagn. Res..

[32] Nayak S.R., Saralaya V., Prabhu L.V., Pai M.M., Vadgaonkar R., D’Costa S. (2007). Pterygospinous bar and foramina in Indian skulls: Incidence and phylogenetic significance. Surg. Radiol. Anat..

[33] Yadav A., Kumar V., Niranjan R. (2014). Pterygospinous bar and foramen in the adult human skulls of North India: Its incidence and clinical relevance. Anat. Res. Int..

[34] Kamath K., Kuberappa V. (2014). Anatomical study of pterygospinous and pterygoalar bar in human skulls with their phylogeny and clinical significance. J. Clin. Diagn. Res..

[35] Goyal N., Jain A. (2016). An anatomical study of the pterygospinous bar and foramen of Civinini. Surg. Radiol. Anat..

[36] Shaw J.P. (1993). Pterygospinous and pterygoalar foramina: A role in the etiology of trigeminal neuralgia?. Clin. Anat..

[37] Ryu S.J., Park M.K., Lee U.Y., Kwak H.H. (2016). Incidence of pterygospinous and pterygoalar bridges in dried skulls of Koreans. Anat. Cell Biol..

[38] Antonopoulou M., Piagou M., Anagnostopoulou S. (2008). An anatomical study of the pterygospinous and pterygoalar bars and foramina–their clinical relevance. J. Cranio Maxillofac. Surg..

[39] Rosa R.R., Faig-Leite H., Faig-Leite F.S., Moraes L.C., Moraes M.E.L., Filho E.M. (2010). Radiographic study of ossification of the pterygospinous and pterygoalar ligaments by the Hirtz axial technique. Acta Odontol. Latinoam..

[40] Chouké K.S. (1947). On the incidence of the foramen of Civinini and the porus crotaphitico-buccinatorius in American whites and negroes; observations on 2745 additional skulls. Am. J. Phys. Anthropol..

[41] Tebo H.G. (1968). The pterygospinous bar in panoramic roentgenography. Oral Surg. Oral Med. Oral Pathol..

[42] Krmpotić-Nemanić J., Vinter I., Hat J., Jalsovec D. (1999). Mandibular neuralgia due to anatomical variations. Eur. Arch. Otorhinolaryngol..

[43] Henry B.M., Pękala P.A., Frączek P.A., Pękala J.R., Natsis K., Piagkou M., Tomaszewski K.A., Tomaszewska I.M. (2020). Prevalence, morphology, and morphometry of the pterygospinous bar: A meta-analysis. Surg. Radiol. Anat..

[44] Das S., Paul S. (2007). Ossified pterygospinous ligament and its clinical implications. Bratisl. Lek Listy.

[45] Shinde V.S., Mallikarjun M., Patil R. (2011). A study on an ossified pterygospinous ligament. J. Clin. Diagn. Res..

[46] Natsis K., Piagkou M., Skotsimara G., Totlis T., Apostolidis S., Panagiotopoulos N.A., Skandalakis P. (2014). The ossified pterygoalar ligament: An anatomical study with pathological and surgical implications. J. Cranio Maxillofac. Surg..

[47] Pękala P.A., Henry B.M., Pękala J.R., Frączek P.A., Taterra D., Natsis K., Piagkou M., Skrzat J., Tomaszewska I.M. (2017). The pterygoalar bar: A meta-analysis of its prevalence, morphology and morphometry. J. Craniomaxillofac. Surg..

[48] Ossenberg N.S. (1970). The Influence of Artificial Cranial Deformation on Discontinuous Morphological Traits. Am. J. Phys. Anthropol..

[49] Kier E.L. (1966). Embryology of the normal optic canal and its anomalies. An anatomic and roentgenographic study. Investig. Radiol..

[50] Asahara H., Inui M., Lotz M.K. (2017). Tendons and Ligaments: Connecting Developmental Biology to Musculoskeletal Disease Pathogenesis. J. Bone Miner. Res..

[51] Zhang Q., Zhou D., Wang H., Tan J. (2020). Heterotopic ossification of tendon and ligament. J. Cell Mol. Med..

[52] Osakabe T., Hayashi M., Hasegawa K., Okuaki T., Ritty T.M., Mecham R.P., Wachi H., Seyama Y. (2001). Age- and gender-related changes in ligament components. Ann. Clin. Biochem..

[53] Iwasawa T., Iwasaki K., Sawada T., Okada A., Ueyama K., Motomura S., Harata S., Inoue I., Toh S., Furukawa K.I. (2006). Pathophysiological role of endothelin in ectopic ossification of human spinal ligaments induced by mechanical stress. Calcif. Tissue Int..

[54] Lang J. (2001). Skull Base and Related Structures: Atlas of Clinical Anatomy.

[55] Lang J. (1977). Structure and postnatal organization of heretofore uninvestigated and infrequent ossifications of the sella turcica region. Acta Anat..

[56] Esen K., Özgür A., Balcı Y., Ten B. (2022). Pterygospinous and pterygoalar bars in children. Surg. Radiol. Anat..

[57] Cunningham C., Scheuer L., Black S. (2016). Developmental Juvenile Osteology.

[58] Frazer J.E. (1920). The Anatomy of the Human Skeleton.

[59] Wood-Jones F. (1931). The Non-metrical Morphological Characters of the Skull as Criteria for Racial Diagnosis: Part I: General Discussion of the Morphological Characters Employed in Racial Diagnosis. J. Anat..

[60] Erdogmus S., Pinar Y., Celik S. (2009). A cause of entrapment of the lingual nerve: Ossified pterygospinous ligament–a case report. Neuroanatomy.

[61] Peuker E.T., Fischer G., Filler T.J. (2001). Entrapment of the lingual nerve due to an ossified pterygospinous ligament. Clin. Anat..

[62] Nayak S.R., Rai R., Krishnamurthy A., Prabhu L.V., Ranade A.V., Mansur D.I., Kumar S. (2008). An unusual course and entrapment of the lingual nerve in the infratemporal fossa. Bratisl. Lek Listy.

[63] Chouké K.S., Hodes P.J. (1951). The ptergoalar bar and its recognition by roentgen methods in trigeminal neuralgia. Am. J. Roentgenol. Radium Ther..

